# SUMOylation of PDGF receptor α affects signaling via PLCγ and STAT3, and cell proliferation

**DOI:** 10.1186/s12860-023-00481-6

**Published:** 2023-05-16

**Authors:** Kehuan Wang, Natalia Papadopoulos, Anahita Hamidi, Johan Lennartsson, Carl-Henrik Heldin

**Affiliations:** 1 Department of Medical Biochemistry and Microbiology, Uppsala University, Uppsala, Box 582, Sweden; 2 Department of Pharmaceutical Biosciences, Uppsala University, Uppsala, Sweden

**Keywords:** PDGFRα, Receptor tyrosine kinase, SUMO, SUMOylation

## Abstract

**Background:**

The platelet-derived growth factor (PDGF) family of ligands exerts their cellular effects by binding to α- and β-tyrosine kinase receptors (PDGFRα and PDGFRβ, respectively). SUMOylation is an important posttranslational modification (PTM) which regulates protein stability, localization, activation and protein interactions. A mass spectrometry screen has demonstrated SUMOylation of PDGFRα. However, the functional role of SUMOylation of PDGFRα has remained unknown.

**Results:**

In the present study, we validated that PDGFRα is SUMOylated on lysine residue 917 as was previously reported using a mass spectrometry approach. Mutation of lysine residue 917 to arginine (K917R) in PDGFRα substantially decreased SUMOylation, indicating that this amino acid residue is a major SUMOylation site. Whereas no difference in the stability of wild-type and mutant receptor was observed, the K917R mutant PDGFRα was less ubiquitinated than wild-type PDGFRα. The internalization and trafficking of the receptor to early and late endosomes were not affected by the mutation, neither was the localization of the PDGFRα to Golgi. However, the K917R mutant PDGFRα showed delayed activation of PLC-γ and enhanced activation of STAT3. Functional assays showed that the mutation of K917 of PDGFRα decreased cell proliferation in response to PDGF-BB stimulation.

**Conclusions:**

SUMOylation of PDGFRα decreases ubiquitination of the receptor and affects ligand-induced signaling and cell proliferation.

**Supplementary Information:**

The online version contains supplementary material available at 10.1186/s12860-023-00481-6.

## Introduction

Signaling by members of the platelet-derived growth factors (PDGF) family, consisting of dimeric isoforms of A-, B-, C- and D-polypeptide chains, has been implicated in embryonal development, wound healing, and tissue homeostasis in the adult, as well as in cancer [1]. PDGF isoforms bind to α- and β-tyrosine kinase receptors (PDGFRα and PDGFRβ, respectively), promoting dimerization of receptors, which in turn causes receptor autophosphorylation, followed by ubiquitination and internalization from the cell membrane. PDGF-AA specifically induces α-receptor homodimers, while PDGF-BB induces both α- and β-receptor homodimers, as well as an α/β receptor heterodimer. Activation of PDGF receptors initiates downstream signaling pathways promoting cell migration and proliferation.

The activity, stability and subcellular localization of proteins are controlled by different posttranslational modifications (PTMs), including phosphorylation on serine/threonine or tyrosine residues, acetylation, methylation, ubiquitination, SUMOylation, and others [2, 3]. Ubiquitination of proteins can occur by addition of single ubiquitin molecules (mono-ubiquitination), or by addition of poly-ubiquitin chains linked via different lysine residues in ubiquitin [4]. In addition, proteins can be modified by certain ubiquitin-like molecules, including five SUMO isoforms, i.e., SUMO1, 2, 3, 4 or 5 [5]. SUMO1 and SUMO2/3 are ubiquitously expressed and conjugated to target proteins differently. Thus, SUMO2/3 can form poly-SUMOylation chains, while SUMO1 takes part in mono-SUMOylation of proteins and is able to terminate SUMO2/3 polymers [6, 7]. Several receptor tyrosine kinases have been reported to be the substrates of the SUMOylation machinery [8–10]. Both ubiquitination and SUMOylation play important roles in the regulation of protein stability, localization, activation and interaction; however, the conjugation of ubiquitin and SUMO to the same protein may lead to different consequences. For example, ubiquitination often induces protein degradation, while SUMO may stabilize its target proteins [11].

PDGFRα and PDGFRβ activate similar signaling pathways, but certain differences have been reported [12, 13]. Both receptors are able to activate ERK1/2 MAP-kinase, albeit with different kinetics and efficiency [14], as well as phosphatidylinositol 3´-kinase (PI3K), Src and phospholipase C-γ (PLC-γ) pathways [15]. It has been reported that PDGFRα is stored inactive in the cytoplasm, likely in Golgi-derived vesicles, while trafficking to cilia is necessary for controlled activation of PDGFRα signaling [16]. In contrast, PDGFRβ is able to signal continuously from the plasma membrane [17] and the intact PDGFRβ is also translocated to the nucleus where it affects chromatin remodeling [18]. The activation, internalization, stability and signaling properties of PDGFRs has been shown to be regulated by phosphorylation and ubiquitination [1, 19]. No SUMOylation has been reported for PDGFRβ, whereas PDGFRα has been demonstrated to be SUMOylated in a mass spectrometry screen [20, 21]. The purpose of this work was to explore the role of SUMOylation in the signaling, stability and subcellular localization of PDGFRα.

## Results

### PDGFRα is SUMOylated

**Fig. 1:**
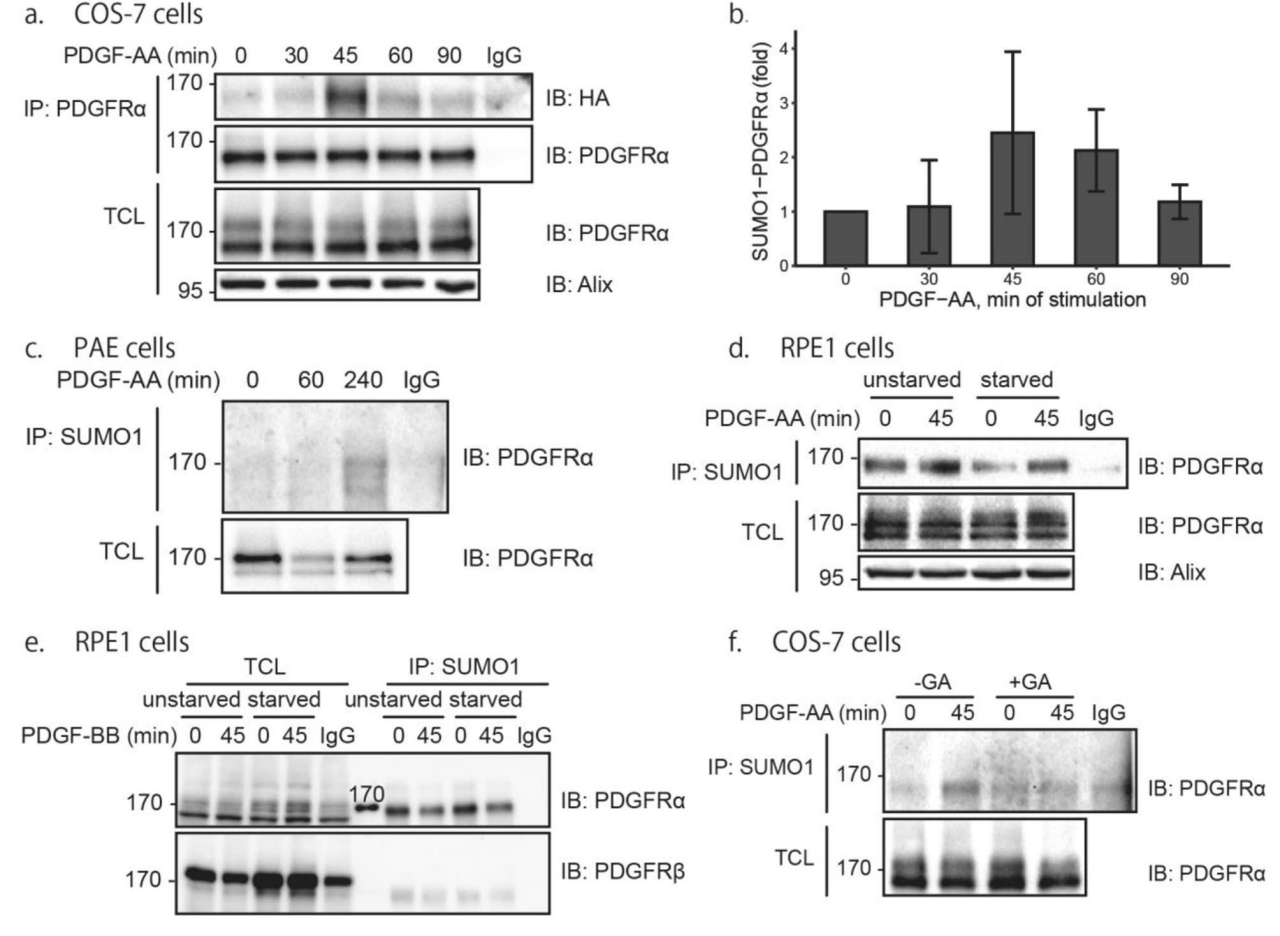
PDGFRα is SUMOylated. (**a**) COS-7 cells were co-transfected with indicated plasmids, followed by serum-starvation for 24 h. Cells were stimulated with 20 ng/ml PDGF-AA or PDGF-BB for different time periods, lysed and subjected to immunoprecipitation using an antibody against PDGFRα, followed by immunoblotting using anti-HA and anti-PDGFRα antibodies. Non-immune IgG was used as a negative control. Total cell lysates (TCL) were immunoblotted with an anti-PDGFRα antibody. HP95 (Alix) was used as a loading control. **(b)** Quantification of SUMOylated PDGFRα in panel A after different times of stimulation of PDGF-AA. Four independent experiments were performed and the standard deviation is indicated. **(c)** PAE cells with stably transfected PDGFRα were stimulated with 20 ng/ml PDGF-AA for 0, 1 and 4 h after serum-starvation for 24 h. Immunoprecipitation of SUMO1 was performed and SUMOylation of PDGFRα was determined by immunoblotting with a PDGFRα antibody. **(d)** RPE1 cells were stimulated with 20 ng/ml PDGF-AA for 0 and 45 min after serum-starvation or the presence of 10% FBS for 24 h. After immunoprecipitation with a SUMO1 antibody, samples were subjected to immunoblotting with a PDGFRα antibody. Receptor expression and loading control (Alix) were determined by immunoblotting of total cell lysates. **(e)** RPE1 cells, starved or not, were treated as in panel d, and cell lysates were heated at 95℃ and sonicated before immunoprecipitation with a SUMO1 antibody, followed by immunoblotting with antibodies against PDGFRα and PDGFRβ. **(f)** COS-7 cells transfected with PDGFRα and HA-SUMO1 were serum-starved and treated with 20 µM ginkgolic acid for 24 h before stimulation with PDGF-AA. SUMO1 was precipitated with HA antibody and PDGFRα was determined by immunoblotting. All experiments were performed three times or more except for the experiments shown in panels c and f which were performed two times. The immunoblots were cropped for clarity. Full length blots are presented in Figure S1

In order to validate the finding by a mass spectrometry analysis that PDGFRα is SUMOylated [20], we transiently co-overexpressed PDGFRα and HA-SUMO1 in COS-7 cells and subjected cell lysates to immunoprecipitation with an antibody against PDGFRα, followed by immunoprecipitation with an HA-antibody; SUMOylation of PDGFRα was detected under starvation conditions and peaked at 45 min of stimulation with 20 ng/ml PDGF-AA (Fig. 1a; quantified in **1b**). In PAE cells stably transfected with PDGFRα, the receptor was also found to be SUMOylated, as determined by immunoprecipitation of endogenous SUMO1 and immunoblotting for PDGFRα (Fig. 1c). We then performed co-immunoprecipitation experiments using RPE1 cells that express endogenous PDGFRα and SUMO1. We found that PDGF-AA induced the SUMOylation of PDGFRα, and that more SUMOylation was observed in cell cultures that were not starved (Fig. 1d). To investigate whether the observed SUMOylation was on PDGFRα, or on some other proteins bound to PDGFRα, we boiled the lysates before immunoprecipitation to remove all PDGFR-bound proteins; SUMOylation of PDGFRα was detected also after this treatment, while no SUMO1 modification of PDGFRβ was seen (Fig. 1e). The amount of SUMOylated PDGFRα under denaturing conditions was decreased after 45 min of PDGF stimulation, suggesting that the observed SUMOylation of PDGFRα, as shown in Fig. 1A, may include not only SUMOylated PDGFRα, but also other PDGFRα-bound SUMOylated proteins. The addition of ginkgolic acid (GA), an E1 activating enzyme inhibitor, impaired the SUMOylation of PDGFRα or associated proteins (Fig. 1f).

### Mutation of K917 abolishes SUMOylation of PDGFRα

**Fig. 2:**
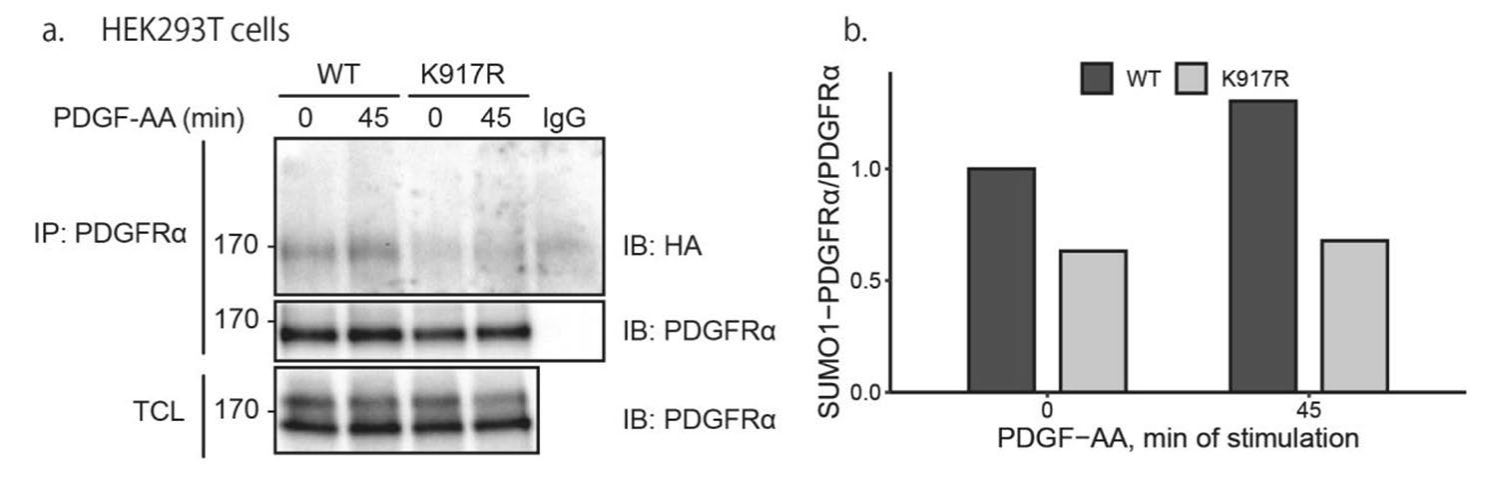
Mutation of lysine residue 917 impairs SUMOylation of PDGFRα. (**a**) HEK293T cells were transfected with WT or K917R mutant PDGFRα and HA-SUMO1. After serum-starvation for 24 h, cells were stimulated with PDGF-AA for 45 min, followed by immunoprecipitation with an anti-PDGFRα antibody. The proteins then were separated by SDS-PAGE and subjected to immunoblotting with indicated antibodies. The expression of PDGFRα was determined by immunoblotting of total cell lysates (TCL). The immunoblots were cropped for clarity. Full length blots are presented in Figure S1. **(b)** SUMOylated PDGFRα was quantified. SUMOylation levels of WT PDGFRα with stimulation of PDGF-AA at 0 min were set as 1. The experiment was performed three times and a representative blot is shown and quantified

Lysine residue 917 is the only reported SUMOylation site in PDGFRα so far [20]. We mutated this residue to an arginine residue (K917R) to investigate if this is indeed an acceptor site for SUMO1. HEK293T cells were transiently co-transfected with wild-type (WT) or K917R mutant PDGFRα and HA-SUMO1. After stimulation with 20 ng/ml PDGF-AA for 45 min under starvation conditions, SUMOylation of WT PDGFRα was determined by immunoprecipitation of PDGFRα, followed by immunoblotting with an HA antibody; significantly less SUMOylation was detected on the K917R mutant as compared to the WT PDGFRα (Fig. 2a; quantified in **2b**). These results suggest that K917 is a main SUMOylation site in PDGFRα, but we cannot exclude that other acceptor sites exist since there was still some SUMOylation left on the mutated receptor.

### SUMOylation at K917 does not affect the stability of PDGFRα

**Fig. 3:**
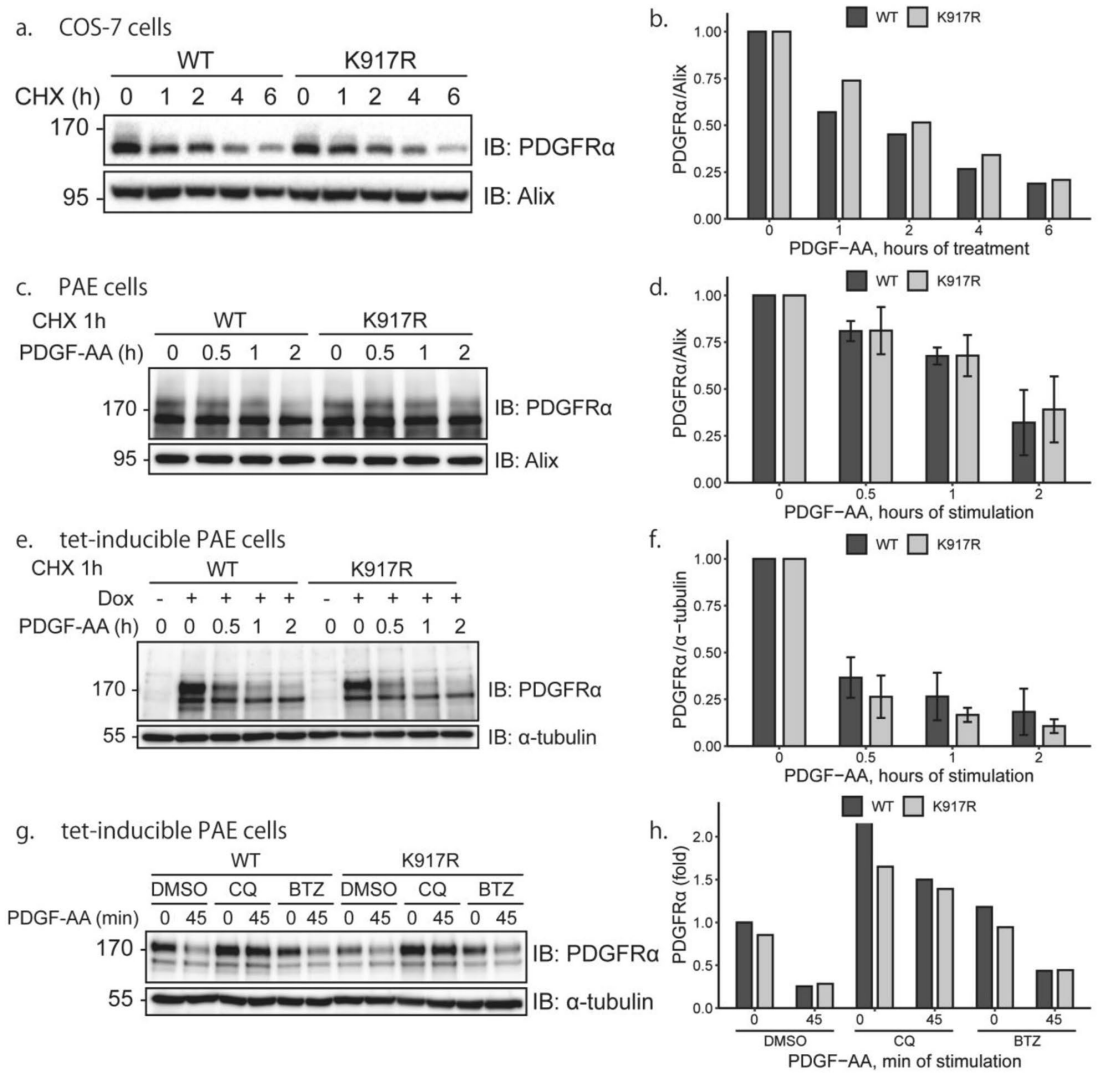
Mutation of lysine residue 917 does not affect the steady state level or ligand-induced degradation of PDGFRα. (**a**) WT or K917R mutant PDGFRα were transfected in COS-7 cells, followed by treatment with 50 µg/ml cycloheximide for 0, 1, 2, 4 and 6 h. The cells were then lysed and immunoblotted with antibodies against PDGFRα and Alix as a loading control. **(b)** The expression of PDGFRα was quantified. WT or K917R mutant PDGFRα levels at 0 h treatment of cycloheximide was set at 1. **(c-f)** WT or K917R mutant PDGFRα were transiently transfected into PAE cells **(c)** or induced with doxycycline in PAE-PDGFRα tet-inducible cell line **(e)**, followed by serum-starvation for 24 h and treatment with 50 µg/ml cycloheximide for 1 h. Cells were then stimulated with PDGF-BB for 0, 0.5, 1 or 2 h, after which the amount of PDGFRα was examined by immunoblotting, and quantified relative to the levels of Alix or α-tubulin which served as loading controls **(d, f)**. The results of three independent experiments were quantified. Expression levels of WT or K917R mutant PDGFRα without PDGF-AA stimulation were set at 1. **(g)** Tet-inducible PAE cells were treated with doxycycline for 48 h to induce the expression of WT or K917R mutant PDGFRα and starved, followed by incubation with 25 µM chloroquine (CQ) or 1 µM bortezomib (BTZ) for 4 h; DMSO served as a control. Cells were then stimulated with 20 ng/ml PDGF-AA for 45 min and lysed for immunoblotting. **(h)** Quantification of the results of panel g. The level of WT PDGFRα without inhibitor treatment and PDGF-AA stimulation was set as 1. All experiments were performed three times or more except for panels a and g which were performed two times. The immunoblots were cropped for clarity. Full length blots are presented in Figure S1

It has been reported that protein SUMOylation may enhance the stability of its substrates by inhibiting its ubiquitination [22, 23]. In order to investigate the effect of SUMOylation on PDGFRα stability, WT or K917R mutant PDGFRα were overexpressed in COS-7 cells. The transfected cells were treated with cycloheximide (CHX) to inhibit the synthesis of new proteins, and after different time periods up to 6 h, cell lysates were prepared and subjected to immunoblotting for PDGFRα (Fig. 3a; quantified in **3b**). No significant difference in the steady state levels of WT and K917R mutant PDGFRα over time was observed. The stability of PDGFRα in response to PDGF-AA stimulation was then investigated in PAE cells transiently transfected with WT or K917R mutant PDGFRα. Upon PDGF-AA stimulation, the levels of WT and K917R mutant PDGFRα were similar (Fig. 3c; quantified in **3d**). In order to make sure that these results were not due to low efficiency of transfection, tet-inducible PAE cell lines were established in which the expression of WT or K917R mutant PDGFRα could be induced by treatment of cells with doxycycline. After induction of PDGFRα expression for 48 h, there was no significant difference of PDGF-AA-induced degradation of WT and mutant PDGFRα (Fig. 3e; quantified in **3f**), which is consistent with the result using transiently transfected PAE cells. PDGF receptors are degraded in lysosomes [24] and proteasomes [19, 25]. Incubation with the lysosomal inhibitor chloroquine (CQ) inhibited the degradation of both WT and K917R mutant PDGFRα to the same extent; the proteasomal inhibitor bortezomib (BTZ) also inhibited the degradation of both WT and K917R mutant PDGFRα slightly, but this was not significant (Fig. 3g; quantified in **3 h**). We observed that the expression levels of the K917R mutant were sometimes lower than the levels of WT PDGFRα, which could be restored with addition of chloroquine, thus suggesting that a fraction of mutated receptor may be incorrectly folded and targeted for lysosomal degradation.

### Mutation of K917 attenuates ubiquitination of PDGFRα

**Fig. 4:**
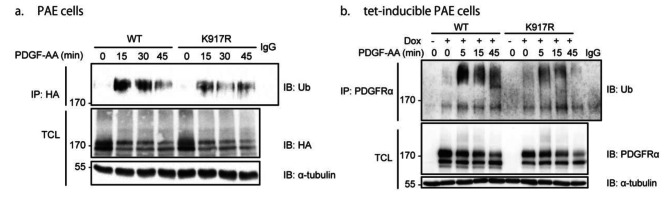
Mutation of lysine residue 917 decreases the ubiquitination of PDGFRα after short-term ligand stimulation. PAE cells transfected with either HA-tagged WT or K917R mutant PDGFRα **(a)**, or tet-inducible PAE-PDGFRα cells induced with doxycycline to overexpress WT or K917R mutant PDGFRα **(b)**, were serum-starved for 24 h and then stimulated with PDGF-AA for indicated time periods. An antibody against HA or PDGFRα was used to immunoprecipitate PDGFRα, followed by immunoblotting with indicated antibodies. Total cell lysates were also subjected to immunoblotting with antibodies against HA, PDGFRα, and α-tubulin which was used as a loading control. Ub, ubiquitin. Each experiment was performed three times or more. The immunoblots were cropped for clarity. Full length blots are presented in Figure S1

We next investigated the impact of SUMOylation of K917 on the ubiquitination of PDGFRα. PAE cells transiently transfected with HA-tagged WT or K917R mutant PDGFRα were subjected to immunoprecipitation with a PDGFRα antibody, followed by immunoblotting with an antibody against ubiquitin. A decrease of ubiquitination of the K917R mutant PDGFRα, compared to the WT receptor, was observed in response to PDGF stimulation (Fig. 4a). Similarly, when WT or K917R mutant PDGFRα was induced in tet-inducible PAE cells, the K917R mutant was less ubiquitinated relative to WT PDGFRα (Fig. 4b).

### Mutation of K917 does not affect the internalization of PDGFRα

**Fig. 5:**
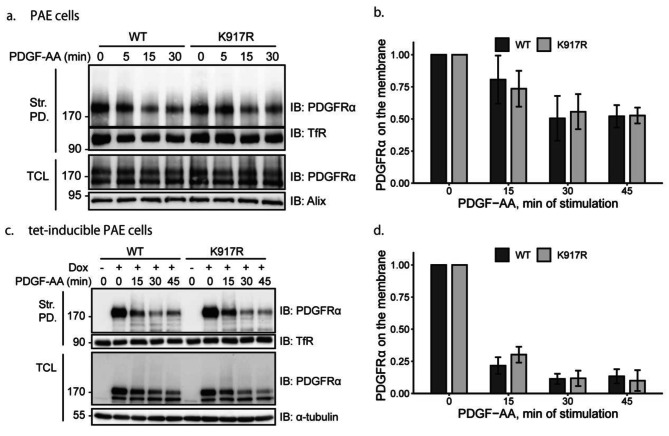
Internalization of PDGFRα from the cell surface is not affected by mutation of lysine residue 917. (**a**) PAE cells were transfected with WT or K917R mutant PDGFRα and serum-starved for 24 h, followed by stimulation with 20 ng/ml PDGF-AA for indicated time periods. Cell surface proteins were then biotinylated. After lysis of cells, biotinylated proteins were collected on streptavidin-agarose beads. Adsorbed proteins and total cell lysates were subjected to SDS-PAGE and immunoblotted with antibodies against PDGFRα, transferrin receptor (TfR) or Alix. Alix was used as a loading control. **(b)** The expression level of PDGFRα remaining on the cell surface compared with transferrin receptor (TfR) from three independent repeats was quantified. The ratio at 0 min of PDGF-AA stimulation was set at 1. **(c)** Expression of WT or K917R mutant PDGFRα in a tet-inducible PAE cell line was induced with doxycycline and assessed as described in panel a. **(d)** The PDGFRα levels on the cell surface relative to TfR on the cell surface from three independent repeats was quantified. The ratio of induced and unstimulated cells was set as 1. The immunoblots were cropped for clarity. Full length blots are presented in Figure S1

Our results indicated that the K917R mutant PDGFRα is less ubiquitinated than WT PDGFRα. Ubiquitination of PDGFRs is linked to internalization of the receptors [19, 26]. SUMOylation has also been reported to affect internalization of its target proteins [27, 28]. In order to investigate whether SUMOylation of PDGFRα affects its internalization, we determined the level of WT and K917R mutant PDGFRα on the cell surface by biotinylating the cell surface receptors after different time periods of stimulation with PDGF-AA. We did not observe any difference in internalization of the K917R mutant PDGFRα as compared to the WT receptor in transfected PAE cells (Fig. 5a; quantified in **5b**). Similarly, the internalization of PDGFRα stably expressed in tet-inducible cell lines was not affected by the K917R mutation (Fig. 5c; quantified in **5d**).

### Mutation of K917 does not affect the localization of PDGFRα to the Golgi apparatus and endosomes

**Fig. 6:**
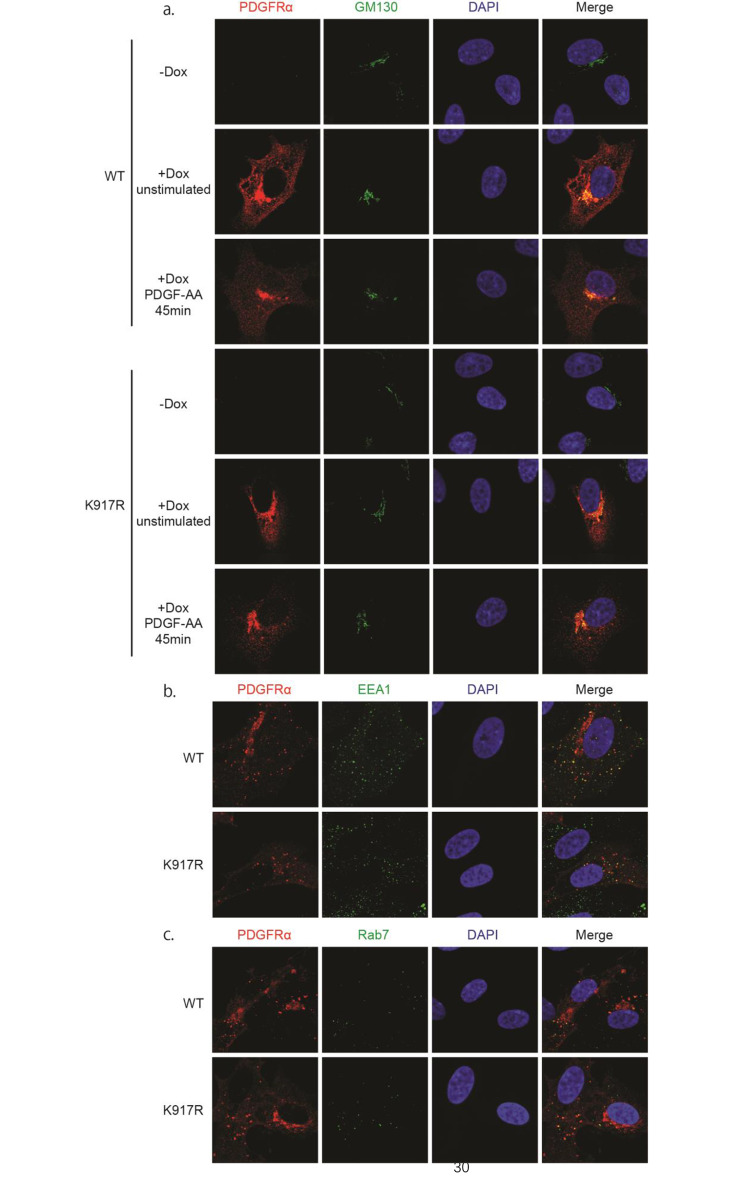
Mutation of lysine residue 917 does not affect the subcellular localization of PDGFRα. (**a**) Tet-inducible PAE cells were grown without doxycycline (-Dox) or treated with doxycycline (+ Dox) to induce the expression of WT or K917R mutant PDGFRα, and then stimulated, or not, with 20 ng/ml PDGF-AA for 45 min, fixed and stained with DAPI and primary antibodies against PDGFRα and the Golgi marker GM130, followed by fluorescent secondary antibodies. **(b, c)** Tet-inducible PAE cells were induced to express WT or K917R mutant PDGFRα, followed by staining with DAPI and with antibodies against PDGFRα, and markers for early (EEA1; **b**) and late (Rab7; **c**) endosomes

SUMOylation of certain RTKs has been shown to affect their subcellular localization [8–10]. In order to investigate whether SUMOylation affects the subcellular localization of PDGFRα, we first determined the localization of WT and K917R mutant PDGFRα to the Golgi in response to PDGF-AA stimulation. Immunofluorescent labeling of PDGFRα and GM130, a marker of Golgi, demonstrated similar localization of WT and K917R mutant PDGFRα at the Golgi apparatus (Fig. 6a). The traffic of WT and K917R mutant PDGFRα to early and late endosomes, in response to stimulation with PDGF-BB, was also investigated by co-staining of PDGFRα with an early endosome marker EEA1 and a late endosome marker Rab7. The results revealed similar localization and WT or K917R mutant PDGFRα to early and late endosomes (Fig. 6b and c).

### Mutation of K917 affects the activation of PLC-γ and STAT3

**Fig. 7:**
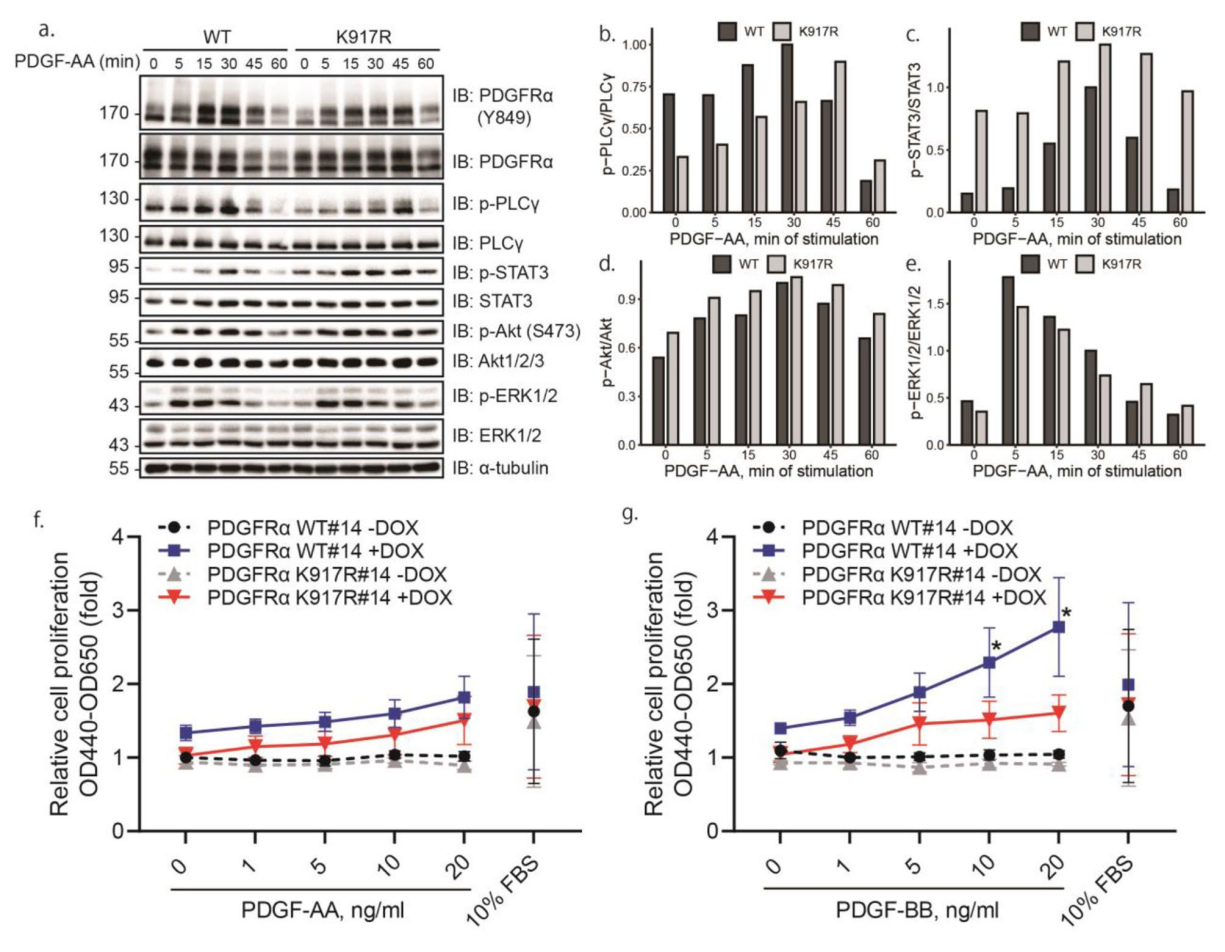
Mutation of lysine 917 affects PDGFRα activation of PLCγ and STAT3 and cell proliferation. (**a**) PAE cells were transiently transfected with WT or K917R mutant PDGFRα, serum-starved and stimulated with 20 ng/ml PDGF-AA for indicated time periods. Expression levels of phosphorylated PLCγ (**b**), STAT3 (**c**), Akt (**d**) and ERK1/2 (**e**) and total proteins were determined using antibodies against phosphorylated PDGFRα (pY849), PLCγ (pY783), STAT3 (pY705), Akt1/2/3 (pS473), ERK1/2 (pThr202/pThr204), and their non-phosphorylated counterparts, as well as α-tubulin as a loading control. The immunoblots were cropped for clarity. Full length blots are presented in Figure S1. **(b-e)** Quantification of phosphorylated proteins relative to total proteins. Phosphorylation at 30 min of stimulation with PDGF-AA was set as 1. The experiment is a representative one out of three experiments performed with similar results. **(f, g)** Tet-inducible PAE cells were seeded into 96 well plates, incubated in Ham’s F-12 media supplemented with 1% FBS, induced or not with doxycycline, and then stimulated with 0, 1, 5, 10 or 20 ng/ml of PDGF-AA **(f)** or PDGF-BB **(g)** for 72 h. Cells incubated in media with 10% FBS were used as a positive control. WST-1 was added into the wells for 4 h and the absorbance of cells incubated in 1% FBS without doxycycline and PDGF ligands was set as 1. The results from four independent repeats are plotted. The statistically significant difference between WT and K917R mutant PDGFRα tet-inducible PAE cell lines was determined by unpaired, two-tail student t-test. *p < 0.05

We then investigated the impact of the mutation of K917 on the downstream signaling pathways induced by PDGFRα activation, using immunoblotting with phospho-specific antibodies. By transiently transfecting PAE cells with WT or K917R mutant PDGFRα, and stimulating the cells with PDGF-AA for different time periods (Fig. 7a), we observed that the phosphorylation of PLC-γ was delayed in cells expressing K917R mutant PDGFRα, peaking at 45 min of stimulation of PDGF-AA (Fig. 7b), and that phosphorylation of STAT3 was enhanced (Fig. 7c). The background phosphorylation of PLCγ in K917R PDGFRα cells, before stimulation with PDGF-AA, was reproducibly decreased compared to WT PDGFRα, whereas the background phosphorylation of STAT3 was increased. This was a consistent finding, the explanation for which remains to be determined. However, activation of other signaling molecules, including ERK1/2 MAP-kinase (Fig. 7d) and Akt, a downstream effector of PI3K (Fig. 7e), were not affected. Similar results were obtained with tet-inducible stable cell lines (data not known).

### Effects of mutation of K917 on cell migration and cell proliferation

It has been reported that SUMOylation may affect cell migration [29–31] and cell proliferation [32, 33]. In view of our finding of altered activation of signaling pathways downstream of PDGFRα, we analyzed whether these changes had functional effects on the cells, but have not found any effect of K917R mutation on PDGFRα-induced migration (data not shown). The effects on cell proliferation in response to different concentrations of PDGF-AA and PDGF-BB, were determined using WT and K917R mutant PDGFRα tet-inducible PAE cell lines. The cells treated with doxycycline responded to both PDGF-AA (Fig. 7f) and PDGF-BB (Fig. 7g) stimulation. The proliferative response of cells expressing K917R mutant PDGFRα to ligand stimulation was inhibited compared with cells expressing the WT receptor, especially when cells were stimulated with PDGF-BB (Fig. 7g).

## Discussion

SUMOylation of PDGFRα at lysine residue 917 was recently reported in a mass spectrometry analysis [20]. In the present study, we confirmed SUMOylation of PDGFRα using immunoblotting, while no SUMOylation of PDGFRβ was detected. Upon PDGF-AA stimulation, SUMOylation of PDGFRα and associated proteins peaked at 45 min when the receptor was overexpressed in COS-7 cells, while phosphorylation and ubiquitination of PDGFRα were seen after 5 min. Introduction of the inhibitor of the SUMO E1 enzyme SAE1/SAE2 ginkgolic acid attenuated SUMOylation of PDGFRα. Interestingly, under denaturing conditions, less SUMOylation was observed upon PDGF-AA stimulation in RPE1 cells, suggesting that, apart from PDGFRα, other proteins binding to PDGFRα may also be SUMOylated. SUMOylation of PDGFRα was detected in RPE1 cells that were not stimulated with PDGF, indicating that some background level of SUMOylation exists in growing cells. It has been found that glucose- and serum-starvation promote REV1 SUMOylation [34]. On the other hand, reduced SF-1 SUMOylation under serum-starvation was reported [35]. Therefore, we investigated the SUMOylation of PDGFRα under serum-starvation versus non-starvation conditions. We observed more SUMOylated PDGFRα under serum-starvation in COS-7 cells (data not shown), while the reverse was true for RPE1 cells. These results indicated that the effects of starvation on PDGFRα SUMOylation may vary in different cell lines.

The crosstalk between SUMOylation and ubiquitination is complex. Protein SUMOylation and ubiquitination can occur on the same lysine residues of their substrates, which might lead to competition between SUMOylation and ubiquitination, in which case SUMOylation may stabilize its target protein by inhibiting its ubiquitination. For example, SUMOylation of lysine residue 21 of IκBα blocks the ubiquitination at the same site, thus stabilizing IκBα [36]. SUMOylation has also been reported to facilitate subsequent ubiquitination of the substrates via STUbLs [37]. In the present study, we noticed a lower degree of ubiquitination of the K917R mutant PDGFRα compared with WT PDGFRα, which may suggest that this site can be both ubiquitinated and SUMOylated. Alternatively, SUMOylation of lysine residue 917 may promote ubiquitination of other lysine residues in the receptor.

SUMOylation has been reported to increase protein stability [22, 38]. Since our results showed less ubiquitination of the K917R mutant PDGFRα, we investigated the degradation and internalization of receptors which are usually promoted by ubiquitination [19]. However, we found no difference in the steady levels or ligand-induced degradation between WT and K917R mutant PDGFRα. Surprisingly, we also did not notice any influence of the K917R mutation on the internalization of the receptor from the cell membrane upon PDGF-AA stimulation, as well as on the trafficking of the PDGFRα to early and late endosomes. SUMOylation has been found to be related to the localization of receptors to the nucleus [8], cilia [39], and Golgi [10]. However, we could not detect any ciliary or nuclear localization of PDGFRα (not shown) and did not notice any difference in the localization between WT and K917R mutant PDGFRα to other organelles.

Posttranslational modifications of RTKs, including ubiquitination and SUMOylation, may affect their downstream signaling. Our study showed delayed activation of PLC-γ and enhanced activation of STAT3 in K917R mutant PDGFRα cells after ligand stimulation. In our previous research, we found that USP17 and USP4 deubiquitinated PDGFRβ and increased the level of activated STAT3 [40]. The present study showed reduced ubiquitination of K917R mutant PDGFRα, which correlated to increased STAT3 activation. This is consistent with the previous finding and suggests that the level of ubiquitination of PDGFRα also may regulate the downstream signaling rather than simply targeting the receptor for proteasomal and/or lysosomal degradation. Since we did not observe any effect of SUMOylation on stability, trafficking or degradation of PDGFRα, it is possible that SUMOylation of PDGFRα affects interactions with downstream effector molecules or their phosphorylation, leading to effects on cell proliferation.

It has been known that abolition of SUMOylation dramatically reduced cell proliferation mediated by Akt1 [33]. Interestingly, we noticed less proliferation of PAE cells induced to express K917R mutant PDGFRα than cells induced to express WT PDGFRα, especially when cells were stimulated with PDGF-BB. The reason for the differences in the proliferative response of wild-type PDGFRα expressing cells to PDGF-AA and PDGF-BB remains to be determined, but could be due to the fact that PDGF-AA is a less potent activator of PDGFRα than PDGF-BB in the PAE cell line. A contribution from endogenous PDGFRβ is unlikely, since PAE cells do not express PDGFRβ and there was no effect of PDGF-BB stimulation on non-induced PAE cells.

In conclusion, by mutating lysine residue 917 of PDGFRα to an arginine residue, we investigated the functional role of SUMOylation of this site on receptor function. We observed some effects on signaling and cell proliferation, however, overall the effects seen were mild. It is possible that there are other SUMOylated residues, in addition to lysine 917, which may have contributed to SUMO-dependent effects in the K917R mutant PDGFRα, possibly involving multiple SUMO enzymes [41].

## Conclusions

In summary, we have identified PDGFRα as a SUMOylation substrate, and have presented an initial characterization of the functional role of SUMOylation of PDGFRα. Further studies on the effect of PDGFRα SUMOylation on receptor regulation and cellular effects remain to be performed.

## Materials and methods

### Reagents

An antibody against PDGFRα (AF 1602) was purchased from R&D Systems (Minneapolis, MN, USA). The antibodies recognizing phospho-PDGFRα (pY849, #3170), phospho-PLCγ1 (pTyr783, #2821), PLCγ1 (#2822), phospho-STAT3 (pTyr705, D3A7, #9145), STAT3 (79D7, #4904), phospho-Akt (pSer473, D9E, #4060), Akt (#9272S), phospho-ERK1/2 (pThr202/pThr204, #9101) and ERK1/2 (137F5, #4695) were from Cell Signaling Technology (Danvers, MA, USA). Antibodies against SUMO1 (sc-5308) and ubiquitin (sc-8017) were from Santa Cruz Biotechnology (Santa Cruz, CA, USA). The α-tubulin antibody (T6074) was from Sigma-Aldrich (St Louis, MO, USA). Primary antibodies against HA (NB600-363) and the transferrin receptor (TfR) (NB100-92243) were from Novus Biologicals (Centennial, CO, USA). Antisera recognizing Alix (HP95) [42] and PDGFRβ (CTβ) [43] were prepared in house. The secondary antibodies used for immunoblotting included goat anti-mouse IgG (62-6520), goat anti-rabbit IgG (65-6120) and rabbit anti-goat IgG (81-1620), were from Invitrogen (Waltham, MA, USA). The inhibitors MG132 (C2211) and chloroquine (C6628) were from Sigma-Aldrich (St Louis, MO, USA). The inhibitor ginkgolic acid (345,887) was purchased from Merck KGaA and bortezomib (#2244) was from Cell Signaling Technology (Danvers, MA, USA).

### Mutagenesis

QuikChange Lightning Site-Directed Mutagenesis Kit (Agilent Technologies, 210,518) was used to mutate lysine residue 917 of PDGFRα to an arginine residue. Thermal cycling for PCR was performed using plasmid pcDNA3-PDGFRα as the template and primers containing the mutation were designed by the online tool (https://www.chem.agilent.com/store/primerDesignProgram.jsp). The amplification products were then digested by *Dpn* I enzyme and transformed into XL10-Gold Ultracompetent Cells provided in the kit following the manual. The plasmids were isolated and sequenced to confirm the presence of mutation. Wild-type (WT) and K917R mutant PDGFRα constructs were further used for transient transfection and to create stable cell lines.

### Cell culture and plasmid transfection

African green monkey kidney fibroblast-like cells COS-7 (purchased from ATCC, CRL-1651), human embryonic kidney (HEK) 293 cells (purchased from ATCC, CRL-1573), and human retinal pigment epithelial-1 (RPE-1) cells (kindly provided by Soren Christensen, University of Copenhagen, Denmark; ATCC, CRL-4000) were cultured in Dulbecco’s modified Eagle medium (DMEM) (Sigma-Aldrich), supplemented with 10% fetal bovine serum (FBS) (Biowest). Porcine aortic endothelial (PAE) cells were cultured in Ham’s F-12 Nutrient Mix, GlutaMAX™ Supplement (Thermo Fisher Scientific) containing 10% FBS. WT and K917R mutant PDGFRα stable inducible cell lines were cultured in DMEM media, supplemented with 0.8 µg/ml puromycin and 10% FBS. For starvation, cells were incubated in DMEM medium supplemented with 0.1% FBS for 24 h. Cells were transiently transfected with plasmids using Lipofectamine 3000 reagent (Invitrogen), following the protocol provided by the vendor.

### Cell lysis, immunoprecipitation and immunoblotting

After various treatments, cells were washed once with phosphate-buffered saline (PBS), pre-chilled at 4℃ and then lysed in RIPA buffer (1% NP-40, 0.5% sodium deoxycholate, 10% glycerol, 20 mM Tris-HCl, pH 7.5, 150 mM NaCl), supplemented with 1 mM Pefa Block, 1 mM sodium orthovanadate (Na_3_VO_4_) and 20 mM N-ethylmaleimide (NEM), on ice for 5 min. The lysates were centrifuged at 13,000 rpm for 15 min at 4℃ and the supernatants were collected. To prepare cell lysates for sodium dodecyl sulfate (SDS)-polyacrylamide gel electrophoresis (SDS-PAGE), 20 µl of 6× SDS sample buffer (500 mM Tris-HCl, pH 6.8, 30% glycerol, 10% SDS, 600 mM dithiothreitol, 0.01% bromophenol blue) was added to 100 µl of the supernatants. For immunoprecipitation, antibodies were added to the supernatants at a final concentration of 0.2 µg/ml and samples were incubated overnight at 4℃ with end-over-end rotation; Dynabeads Protein A/Protein G (Invitrogen) was then added and incubation was prolonged for one more hour. The beads were washed with lysis buffer three times and heated in SDS sample buffer at 95℃ for 3 min, followed by SDS-PAGE and immunoblotting. After electrophoresis, proteins were electro-transferred to polyvinylidene difluoride (PVDF) membranes (Immobilon P). The membranes were blocked in PBS containing 0.1% Tween 20 (PBST) and 5% bovine serum albumin (BSA) for 1 h, incubated with primary antibodies diluted in PBST containing 1% BSA overnight at 4℃, washed three times in PBST for 5 min each time, incubated in peroxidase-conjugated secondary antibodies for 1 h, and then washed three times in PBST. Proteins were visualized by SuperSignal West Dura Extended Duration Substrate (Thermo Scientific) and a charge-coupled device (CCD) camera (Bio-Rad). Image Lab (Bio-Rad) was used to quantify the bands.

### Biotinylation of cell surface PDGFRα

After starvation and stimulation, cells were washed once with pre-chilled PBS, incubated in 0.3 mg/ml EZ-Link Sulfo-NHS-SS-Biotin (Thermo Scientific) in PBS for 1 h on ice, blocked with 50 mM Tris-HCl, pH 7.4 for 5 min, washed once with PBS, and lysed in RIPA buffer for 5 min on ice. After centrifugation at 13,000 rpm for 15 min at 4℃, prewashed Pierce Streptavidin Agarose (Thermo Scientific) was added to the supernatant and incubated end-over-end for 1 h at 4℃, followed by three times washes in lysis buffer and heating at 95^o^C in SDS sample buffer.

### Establishment of tet-inducible cell lines

Lenti-X Tet-One Inducible Expression System (Takara Bio USA) was used to establish tet-inducible cell lines. In-Fusion HD Cloning Kit (Takara Bio USA) was used to clone WT or K917R mutant PDGFRα into the pLVX-TetOne vectors and lentiviral particles were produced using Lenti-X Packaging Single Shots (VSV-G), containing transfection reagent and lentiviral packaging plasmids. 293T cells were transfected with indicated plasmids and lentiviral particles were harvested 24- and 48-hours post-transfection. The collected medium was diluted twice with F-12 medium containing 10% FBS and added to a 6-well plate with PAE cells supplemented with 8 µg/ml polybrene. After 16 h, the medium was replaced with DMEM medium supplemented with 10% FBS and 0.8 µg/ml puromycin. The surviving PAE cells were cultured in medium with puromycin and induced with 100 ng/ml doxycycline to express WT or K917R mutant PDGFRα.

### Cell proliferation assay

Single clones selected from tet-inducible PAE cells were seeded into 96 well plates at a density of 500 cells per well and cultured for 24 h. The media was replaced with 100 µl of F-12 media supplemented with 1% FBS and 1–20 ng/ml PDGF-AA or PDGF-BB in the absence or presence of 100 ng/ml doxycycline, while 10% FBS was used as a positive control. After incubation for 3 days, 10 µl of WST-1 reagent (Roche, 11,644,807,001) was added into the well, incubated at 37℃ in 5% CO_2_ for 4 h. The absorbance was then measured using an ELISA reader at 440 nm, while 650 nm was used as the reference wavelength.

### Statistical analysis

Data were analyzed with Microsoft Excel or GraphPad Prism. The values are shown as mean ± standard deviation for experiments with more than three repeats. The statistical significance was determined by unpaired, two-tail student t-test. *p < 0.05.

## Electronic supplementary material

Below is the link to the electronic supplementary material.

Supplementary Material 1

## Data Availability

The reagents used and datasets analyzed in the current study are available from the corresponding author on reasonable request.
