# Supplementary material for: SUMOylation of PDGF receptor α affects signaling via PLCγ and STAT3, and cell proliferation
Source: BMC Mol Cell Biol. 2023 May 16;24:19. doi: 10.1186/s12860-023-00481-6 (PMC10186711; doi:10.1186/s12860-023-00481-6)
Supplement: Supplementary file 1 — Supplementary Material 1 [file 12860_2023_481_MOESM1_ESM.docx]

**SUMOylation of PDGF receptor α affects signaling via PLCγ and STAT3, and cell proliferation**

Kehuan Wang, Natalia Papadopoulos, Anahita Hamidi, Johan Lennartsson and Carl-Henrik Heldin

**Supplementary Information**

**SUPPLEMENTARY FIGURES**


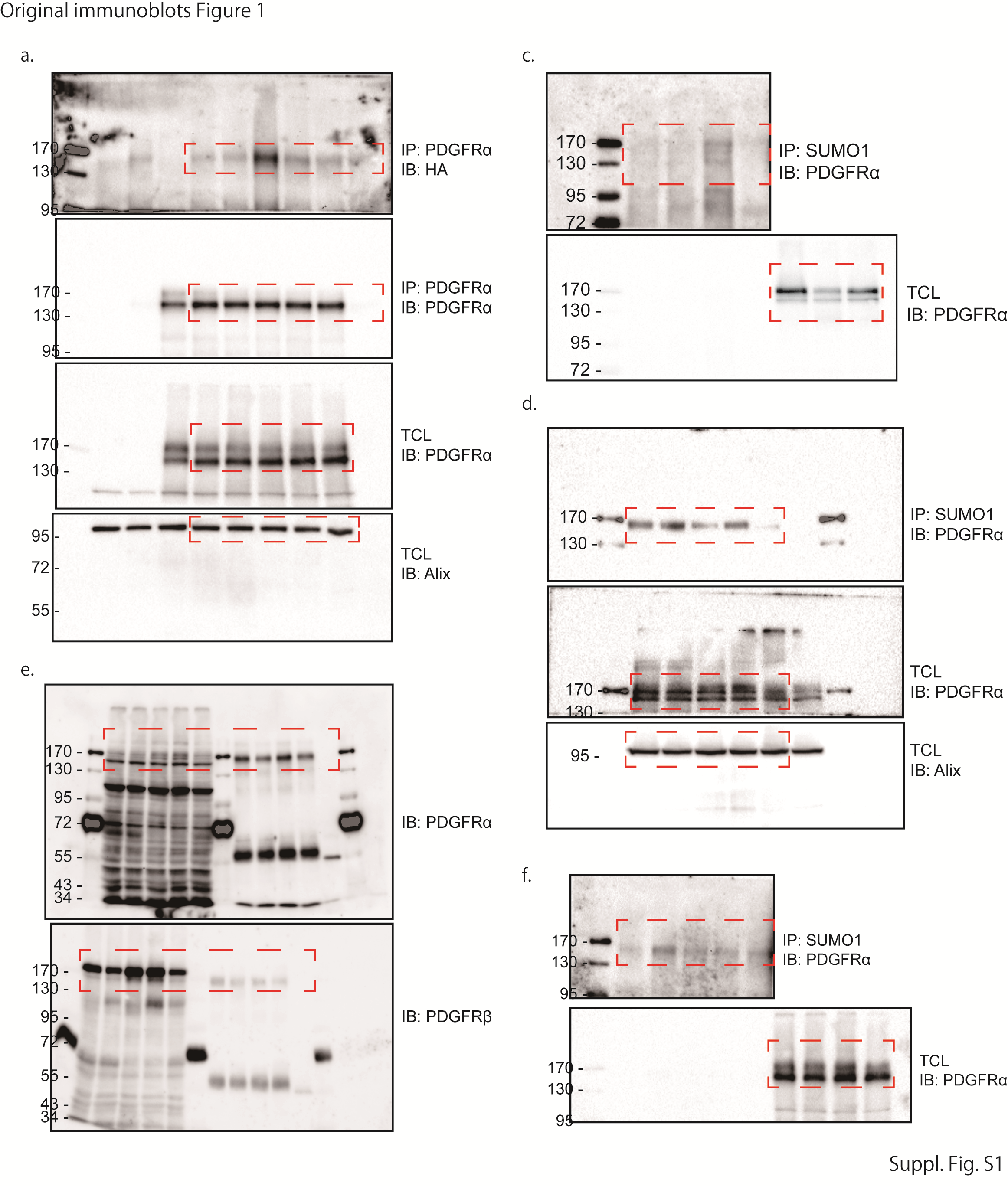

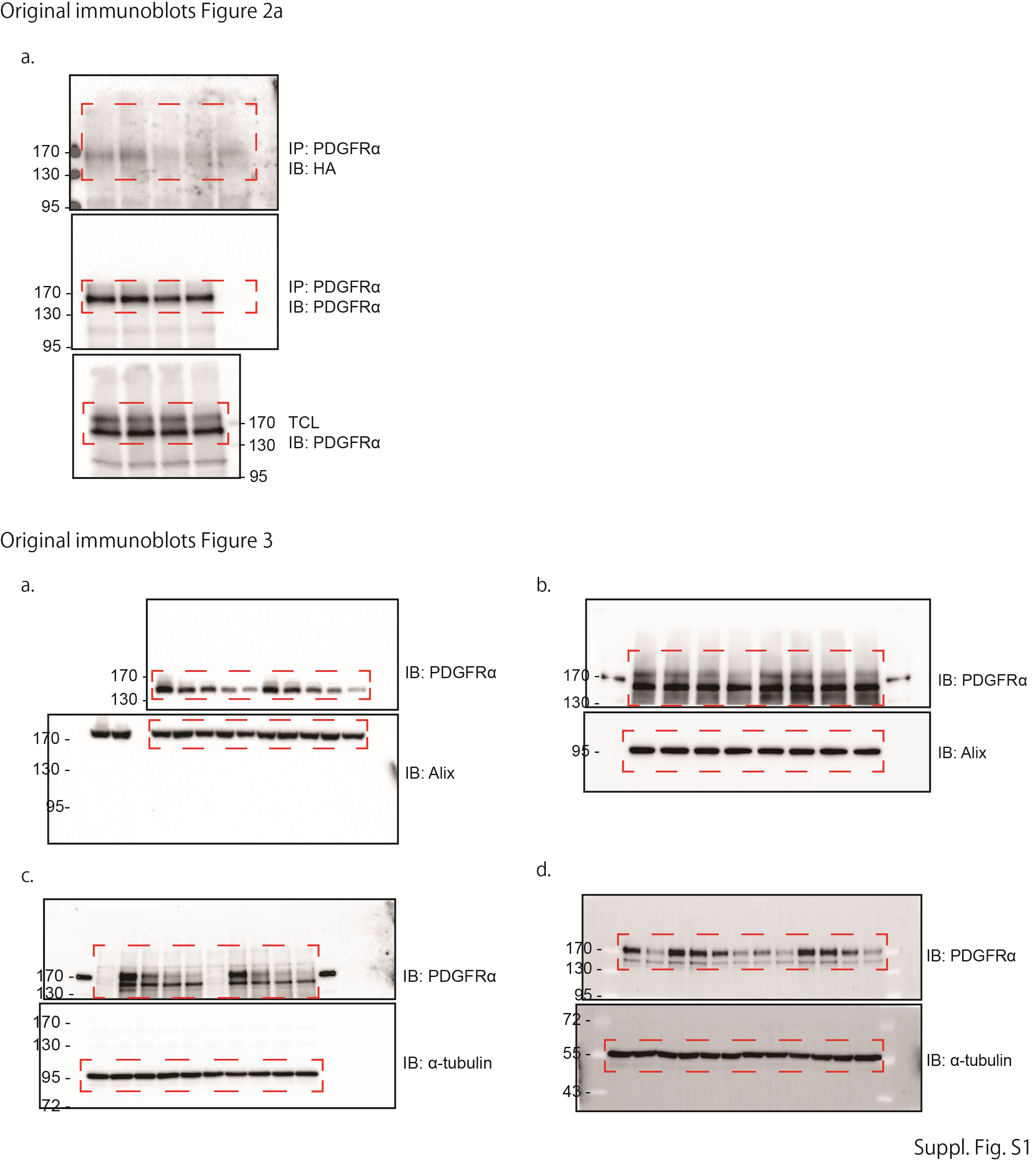

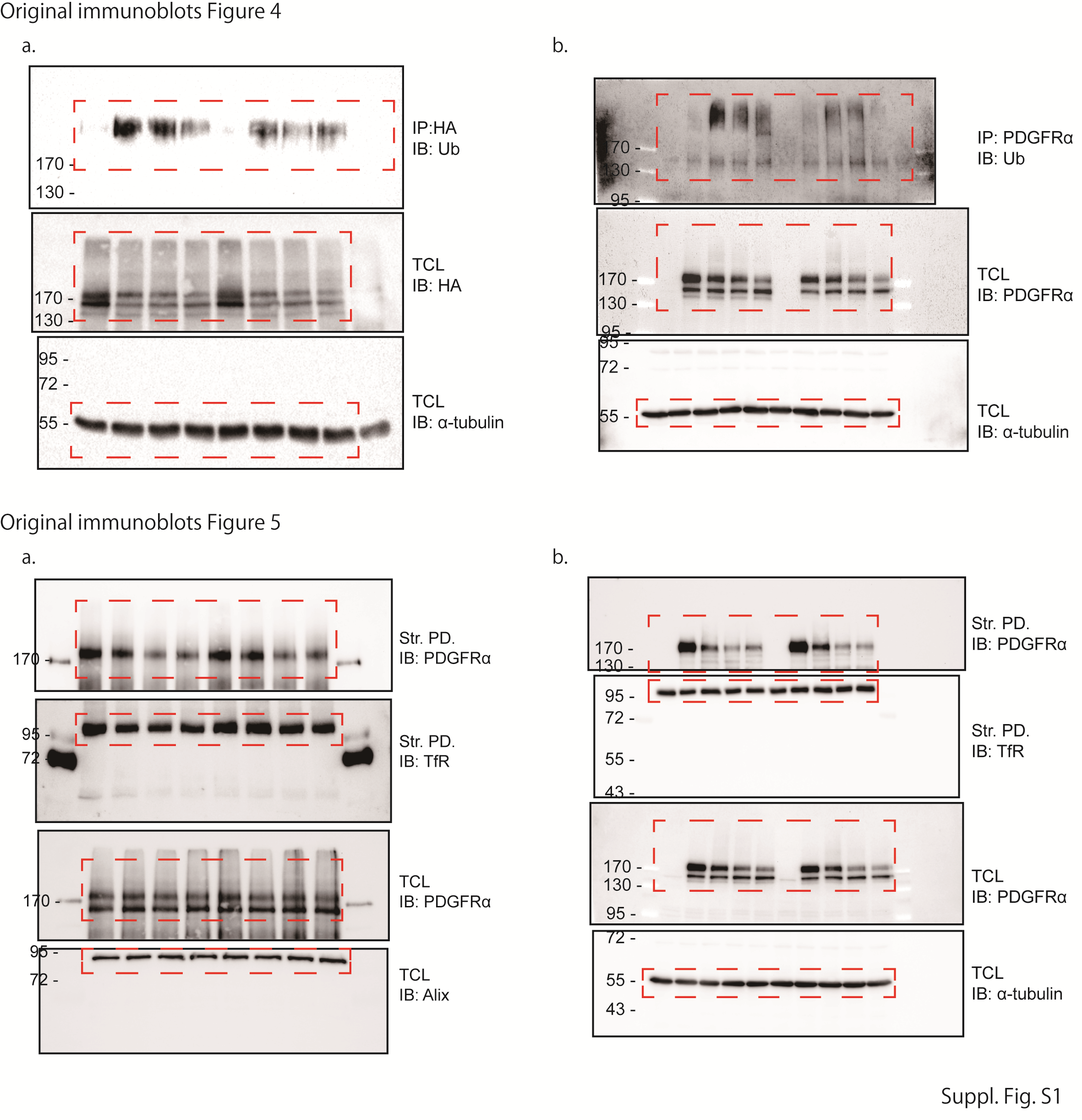

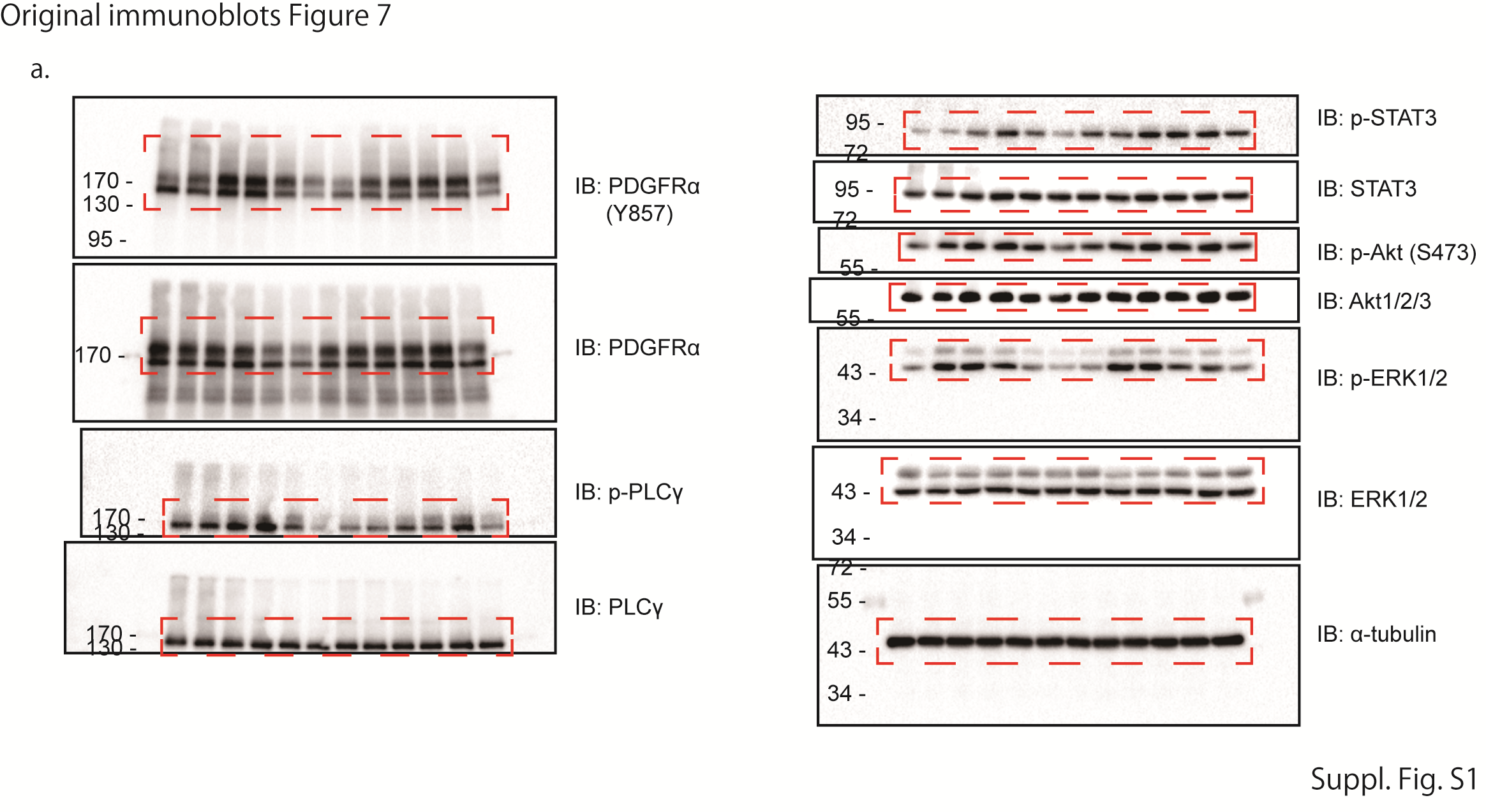


Figure S1. **Original immunoblots.** Uncropped immunoblots determining the expression of the indicated proteins with molecular size markers. The cropped region presented in the main figures were marked with dotted rectangles.
